# Subchondral injection of human umbilical cord mesenchymal stem cells ameliorates knee osteoarthritis by inhibiting osteoblast apoptosis and TGF-beta activity

**DOI:** 10.1186/s13287-025-04366-7

**Published:** 2025-05-09

**Authors:** Congzi Wu, HuiHui Xu, Zhen Wu, Haipeng Huang, Qinwen Ge, Jianbo Xu, Jiali Chen, Pinger Wang, Wenhua Yuan, Hongting Jin, Peijian Tong

**Affiliations:** 1 Institute of Orthopaedics and Traumatology of Zhejiang Province, The First Affiliated Hospital of Zhejiang Chinese Medical University (Zhejiang Provincial Hospital of Chinese Medicine), No.548 Binwen Road, Binwen District, Hangzhou, Zhejiang 310053 PR China; 2 The First School of Clinical Medicine, Zhejiang Chinese Medical University, No.548 Binwen Road, Binwen District, Hangzhou, Zhejiang, 310053, PR China; 3 Department of Orthopaedic Surgery, The First Affiliated Hospital of Zhejiang Chinese Medical University, No.54 Youdian Road, Shangcheng District, Hangzhou, Zhejiang, 310006 PR China; 4 Department of Orthopaedic Surgery, Tongde Hospital of Zhejiang Province, No.234 Gucui Road, Xihu District, Hangzhou, Zhejiang,, 310012 PR China; 5 The First People’s Hospital of Xiaoshan District, Xiaoshan Affiliated Hospital of Wenzhou Medical University, No.199 Shixinnan Road, Xiaoshan District, Hangzhou, Zhejiang, 311200, PR China; 6 Department of Orthopaedic Surgery, Hangzhou Red Cross Hospital (Zhejiang Hospital of Integrated Traditional Chinese and Western Medicine), No.208 East Huancheng Road, Gongshu District, Hangzhou, Zhejiang, 310003, PR China

**Keywords:** Umbilical cord-derived mesenchymal stem cells, Subchondral bone, Apoptosis, Osteoblast, TGF-β signaling

## Abstract

**Background:**

Osteoarthritis (OA) is a common degenerative disease caused by multiple pathological mechanisms wherein subchondral bone malfunction plays a substantial role. Recently, subchondral (SC) injection of orthobiologics has been attracting growing interest albeit the mainstream delivery method of mesenchymal stem cells (MSCs) is through intra-articular (IA). This study investigates the effect of SC injection of human umbilical cord mesenchymal stem cells (UCMSCs) on OA and its possible therapeutic mechanism compared to IA injection.

**Methods:**

Male Sprague-Dawley rats with anterior cruciate ligament transection (ACLT) received saline or UCMSC injections via SC or IA. Consecutive injections once a week for three weeks and withdrawal for another four weeks, followed by Radiographical scanning, histopathological, immunohistochemical, and terminal deoxynucleotidyl transferase (TdT)-mediated dUTP nick-end labelling (TUNEL) staining. Cell counting Kit-8 (CCK-8) assay, alkaline phosphatase (ALP), alizarin red staining (ARS), TUNEL, flow cytometry, quantitative real-time polymerase chain reaction (qRT-PCR) and Western blotting were employed in TNFα-induced MC3T3-E1 cells to illustrate the exact pathogenesis mechanism.

**Results:**

IA and SC UCMSC injections preserved cartilage, synovium, and subchondral bone parameters like trabecular bone volume fraction (BV/TV). SC injection uniquely improved Trabecular separation (Tb.Sp) and Trabecular number (Tb.N). SC and IA injections of UCMSCs demonstrated equivalent efficacy in promoting osteoblastic bone formation and attenuating aberrant angiogenesis of subchondral bone. In addition, we demonstrated that osteoblast apoptosis and Smad2-dependent TGF-beta (TGF-β) are crucial and interactive subchondral bone pathological features in OA. In vivo and vitro studies further revealed that UCMSCs inhibited excessive TGF-β/pSmad2 signaling to regulate aberrant vascularization, osteoblast apoptosis and differentiation imbalance, ultimately maintaining osteochondral homeostasis.

**Conclusions:**

The efficacy of UCMSCs for treating OA rats via SC injection was equivalent to that of IA; and even superior to IA in terms of subchondral bone phenotype via regulating apoptosis and TGF-β/pSmad2 signaling in osteoblasts, suggesting SC injection of UCMSCs as a potential and promising cell therapy for OA treatment.

**Supplementary Information:**

The online version contains supplementary material available at 10.1186/s13287-025-04366-7.

## Background

Osteoarthritis (OA) is a common disorder characterized by articular cartilage degeneration, subchondral bone remodeling, meniscal deterioration, synovial inflammation and aberrant vascularization [1, 2]. Although established risk factors including genetic predisposition, mechanical overload, aging, and obesity are well-documented, the precise pathogenic mechanisms driving OA remain unresolved [3]. The last three decades have seen a growing incidence rate of OA as societies age, with estimated annual percentage change of 0.32% [4, 5]. Currently, over 595 million individuals worldwide lived with OA and numbers are predicted to increase up until 2050, placing a larger strain on the health-system burden globally [6]. Unfortunately, the treatments up to now are mostly symptom-relieving, and disease-modified treatment is still lacking because the exact pathogenesis of OA remains unclear [7].

Although commonly described as a wear-and-tear disease, OA is now recognized as a whole-joint pathology involving multiple tissue components [8]. Emerging evidence highlights the pathological significance of meniscal degeneration and infrapatellar fat pad remodeling in OA progression [9, 10]. Notably, subchondral bone microarchitectural alterations and associated histopathological changes have been identified as equally crucial to articular cartilage degeneration in OA pathogenesis, demonstrating comparable etiological importance [11, 12]. The homeostasis of the subchondral bone relies on coupled bone remodeling, namely osteoblast-mediated bone formation and osteoclast-mediated resorption. Accelerated subchondral bone turnover is seen in the early stages, whereas the subchondral bone sclerosis is observed during the advanced and late stages [13]. However, the high bone turnover leads to insufficient bone mineralization, thus compromising mechanical property of the newly formed bone [14]. Additionally, evidence also shown that H-type vessels with high CD31 expression aggravate subchondral angiogenesis and aberrant osteogenesis [15]. Osteoblast plays a critical role in the maintenance of normal bone remodeling. Although apoptosis is essential to physiological bone turn over, overactivated apoptosis will result in osteoblast dysfunctions, reducing osteoblast number and impairing osteoblast proliferation differentiation [16]. Extensive research has shown that transforming growth factor-beta (TGF-β) signaling plays a pivotal role in regulating homeostasis of both articular cartilage and subchondral bone [17, 18]. Therefore, the best strategies for the treatment of OA should target the multiple pathological alterations in subchondral bone.

Recently, mesenchymal stem cells (MSCs) have gained great attention as a promising candidate in regenerative medicine because of their multiple mechanisms of action, including regenerative effects, multilineage differentiation potential, paracrine effects, immunomodulatory and homing properties [19, 20]. MSCs derived from multiple sources such as bone marrow, adipose tissue and the umbilical cord and can be divided into autologous MSCs and allogeneic MSCs according to cell donor. In comparison with autologous MSCs such as bone marrow-derived MSCs and adipose tissue-derived MSCs, human umbilical cord mesenchymal stem cells (UCMSCs) possess considerable advantages, including better proliferation rates, greater expansion ability, higher purity, and bountiful supply along with no harm to the donor safety, since umbilical cord is typically discarded after birth [21]. Notably, UCMSCs could regulate bone metabolism via paracrine signaling [22], which implies that UCMSCs may also have a regulatory effect on subchondral bone homeostasis. Previous research has initially established that UCMSCs are effective for the treatment of OA in pre-clinical and clinical studies [23–25]. However, much uncertainty still exists about the precise mechanisms and impacts on the development of OA, particularly at the cellular levels of subchondral bone.

Currently, the mainstream intervention modalities of MSCs are intra-articular (IA) injection or surgical implantation [26]. While IA injection of MSCs do alleviate pain and improve function, the effect of this therapy on preventing OA progression is controversial [27, 28]. In contrast, recent data suggest that injections of MSCs into the subchondral bone may be superior to IA injections for the management of OA [29]. Based on the assumption that subchondral bone, as a therapeutic target prior to overlying articular surface, is abnormal (with a decrease of MSCs) and bone marrow lesions in subchondral bone is closely related to prognosis of OA patients [30]. However, research to date has not yet nail down the precise mechanisms behind OA by subchondral (SC) injection. Therefore, this study set out to investigate the effect of SC bone injection of UCMSCs on OA and its possible therapeutic mechanism compared to IA injection. We conjectured that SC injection of UCMSCs could ameliorate OA by inhibiting osteoblast apoptosis and TGF-β activity.

## Materials and methods

### Human UCMSCs preparation and identification

UCMSCs were purchased from S-Evans Biosciences Co., Ltd., Zhejiang, China. The cells were cultured in Alpha modified Eagle’s medium (αMEM )(Gibco, MD, United States) supplemented with 10% fetal bovine serum (FBS) (Sigma, MO, United States) at 37℃ in 5% CO_2_ as previous described [31]. Cells at passage 2–6 were used for experiments. The immunophenotyping of UCMSCs were examined by Fluorescence activated Cell Sorting (FACS) analyses. UCMSCs were trypsinized and suspended in BD Pharmingen stain buffer, then the cells (10^6^ cells/ml) were incubated at 4 °C in dark for 20 min with antibodies of CD29-PB450 (BD Biosciences, 568994; 1:20), CD90-APC (BD Biosciences, 561971; 1:20), CD45-FITC (BD Biosciences, 555482; 1:5), and CD34-PE (BD Biosciences, 550761; 1:5). After washed and resuspended i n 100 µL stain buffer, the cell surface marker expression of UCMSCs were analyzed via flow cytometer (BD Accuri C6, NJ, United States). Flow cytometry data were acquired using CytExpert Software (v2.4.0.28, Beckman Coulter). To assess multilineage differentiation potential, UC-MSCs were cultured in osteogenic, adipogenic, and chondrogenic induction media (Thermo Fisher Scientific, USA) per manufacturer protocols. Differentiation was confirmed through Alizarin Red (calcium), Oil Red O (lipid droplets), and Alcian Blue (proteoglycans) staining (Beyotime, Shanghai, China).

### Anterior cruciate ligament transection (ACLT) rat model and UCMSCs treatment

**Fig. 1:**
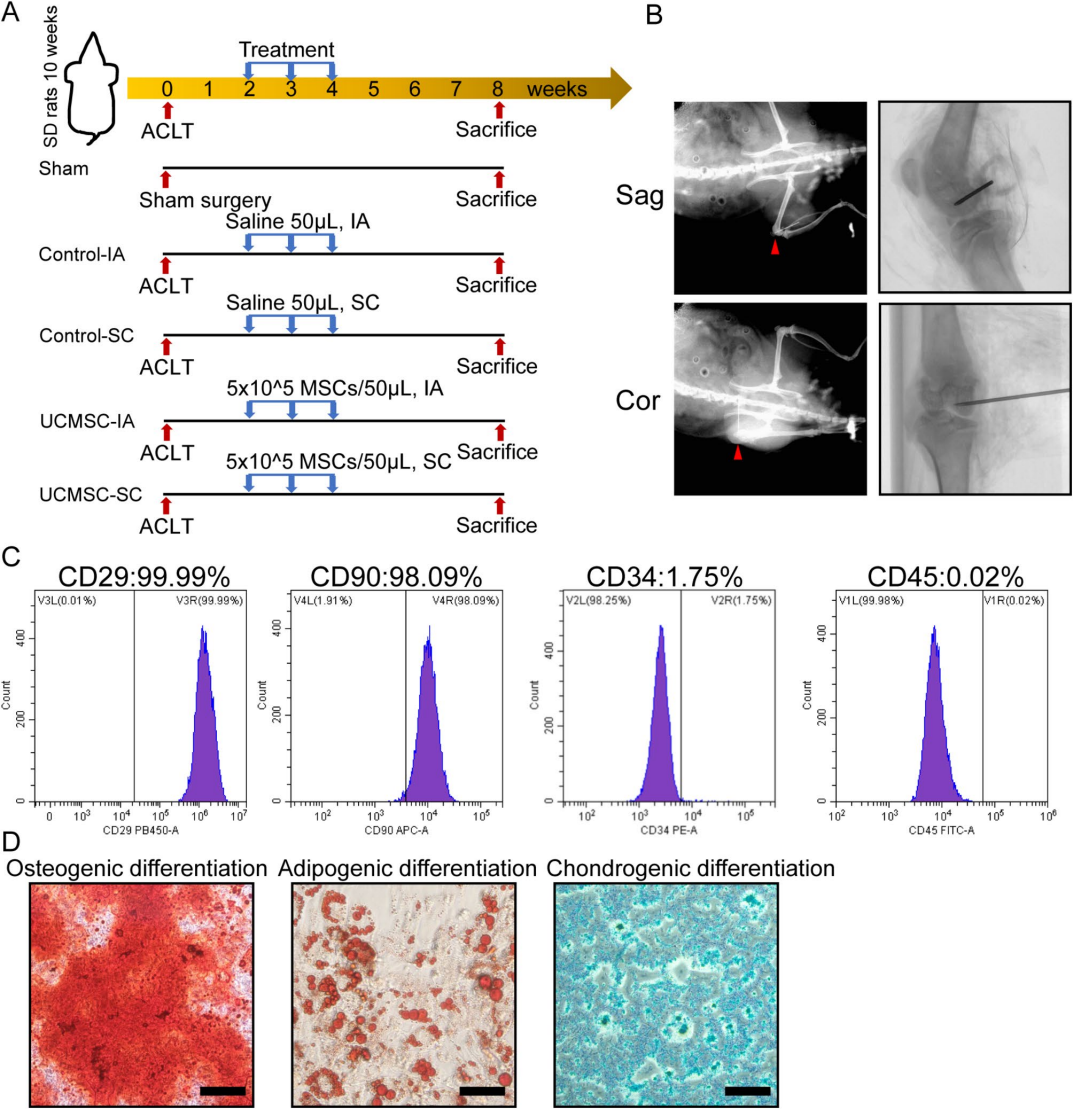
Characterization of UCMSCs and Study design. (**A**) Schematic graph of the study design. Male SD rats were performed ACLT followed by intra-articular or subchondral bone injection with saline or UCMSCs at 2, 3, 4 weeks and sacrificed at 8 weeks. (**B**) Representative x-ray images of UCMSCs injective position. Red arrow indicated injection site. (**C**) Flow cytometry analysis of specific surface markers of UCMSCs. ACLT: anterior cruciate ligament transection; IA: intra-articular injection; SC: subchondral bone injection; Sag: sagittal position; Cor: coronal position. (**D**) Representative images of osteogenic (Alizarin red S staining), adipogenic (Oil Red O staining) and chondrogenic (Alcian Blue staining) differentiation assay. Scale bar, 200 μm

The procedures of this study were approved by the Experimental Animal Ethics Committee of Zhejiang Chinese Medical University on October 10, 2022 (No. 20221010-13) and conducted complied with the ARRIVE guidelines 2.0 and NIH Guide for the Care and Use of Laboratory Animals. Forty male Sprague-Dawley rats (300 ± 20 g) at 10 weeks of age were purchased from Zhejiang Chinese Medical University Laboratory Animal Research Center. All rats were housed at a constant room temperature of 23 ± 2 °C with a 12:12 light/dark cycle and free access to water and food in a specific pathogen-free (SPF) condition. An ACLT model was constructed on the right knee joints as reported previously [32,33]. Briefly, after narcotized with 3% pentobarbital sodium (40 mg/kg, i.p.), the rat’s knee joint capsule was opened with the patellar dislocated laterally. Then, the anterior cruciate ligament was fully cut using a micro-scissor. The anterior drawer test was adopted to verify the success of ACLT. In the sham group, rats were anesthetized, and the knee joint capsule were opened without hurting anterior cruciate ligament. Two weeks after the surgery, the rats were randomly divided into five groups: (1) Sham group (*n* = 6), (2) Control-IA group (performed ACLT and IA injected 50 µL saline once a week for three weeks, *n* = 8), (3) Control-SC group (performed ACLT and SC injected 50 µL saline once a week for three weeks, *n* = 8), (4) UCMSC-IA group (performed ACLT and IA injected 5 × 10^5^ UCMSCs in 50 µL saline once a week for three weeks, *n* = 9), and (5) UCMSC-SC group (performed ACLT and SC injected 5 × 10^5^ UCMSCs in 50 µL saline once a week for three weeks, *n* = 9) (Fig. 1A, B). Rats were anesthetized during IA or SC injection. Sample size was determined via the resource equation method [34]. A 15% attrition buffer was added to ensure robust detection of intergroup differences while controlling type I error. The SC injections were administered under radiographic guidance as previously reported [35]. A 26-gauge needle was used to perforate the cortical bone, followed by insertion of a 29-gauge microinjection needle (from the same batch as those used for IA injections) to minimize trabecular damage during infusion.

### Radiographic (X-ray) and Micro-CT scanning (µCT)

Four weeks after the final UCMSCs injection, rats were euthanized by CO₂ inhalation, and their knee joints samples were harvested and fixed with 4% paraformaldehyde (PFA) for 72 h at 4 °C. The sagittal images were captured by the X-ray (Carestream FX Pro, NY, United States). After that, data were collected using a high-resolution µCT scanner (Skyscan 1275, Bruker, Kontich, Belgium) with scanning parameters of 11 μm per pixel resolution, 65 kV voltage, and 80 µA current. The images were reconstructed using NRecon v1.6 (Bruker, Kontich, Belgium). Then, the images of the subchondral bone of the medial tibial plateau for quantitation were obtained by drawing a region of interest (ROI) using CTAn v1.9 (Bruker, Kontich, Belgium), through which Bone parameters were analyzed, including trabecular number (Tb·N), trabecular bone volume fraction (BV/TV), trabecular thickness (Tb·Th), and trabecular separation (Tb·Sp). CTVol v3.0 (Bruker, Kontich, Belgium) were used for visualization.

### Histopathological and Immunohistochemical/immunofluorescence analyses

After decalcified in 14% ethylenediamine tetra acetic acid (EDTA) (PH 7.4) for 2 months, rat knee joints were embedded in paraffin and sectioned at 4 μm. The sections were subsequently stained with Alcian blue hematoxylin/Orange G (ABH/OG), immunohistochemistry (IHC) and immunofluorescence (IF). The histopathology of cartilage degeneration in the tibial plateaus was scored by three blinded evaluators using the OARSI system [36]. The inflammation of synovium were examined by using the synovitis score [37]. Quantitative measurement of Masson staining was according to the method of previous reports [38]. For IHC, replicates of paraffin sections were incubated for 4 h at 60 ℃ in 0.01 M citrate buffer (Solarbio, Beijing, China) for antigen retrieval after deparaffinage and rehydration. Next, the sections were incubated with primary antibodies against Collagen II (Col2; Abcam, ab34712; 1:200), Aggrecan (ACAN; Bioss, bs-11655R; 1:200), MMP13 (Abcam, ab39012; 1:200), Adamts5 (Bioss, bs-3573R; 1:200), Osteocalcin (OCN; Serviobio, GB11233; 1:300), CD31 (Arigo, ARG52748; 1:100), TGF beta 1 (TGFβ1) (Proteintech, 21898-1-AP; 1:300), and pSmad2 (Thermofisher, 44-244G; 1:300) overnight at 4 ℃, followed by applying with the secondary antibody (PV9001; ZSGQ-BIO, Beijing, China) for 20 min at room temperature. Then the slides were stained with a diaminobenzidine (DAB) solution (ZLI-9019; ZSGQ-BIO, Beijing, China) and hematoxylin. For IF, samples were incubated with a primary antibody against IL-1β (Abcam, ab254360; 1:200) overnight at 4 °C, followed by incubation with a fluorescent secondary antibody (Thermo Fisher Scientific, A31572; 1:1000) in the dark for 1 h at room temperature. Nuclear staining was performed using DAPI (Solarbio, C0065; 1:1000). The positive staining was captured with a light microscope (Axioscope A1; Zeiss, Oberkochen, Germany) and the semi-quantification was assessed by the ImageJ software (National Institutes of Health, Bethesda, MD, USA).

### Terminal deoxynucleotidyl transferase (TdT)-mediated dUTP nick-end labelling (TUNEL) assay

The TUNEL Assay Kit (C1088, Beyotime, Shanghai, China) was used to measure apoptosis in rat knee joint samples. Briefly, after being dewaxed and rehydrated, sections were incubated in TUNEL assay solution at 37 °C for 30 min, followed by DAPI for 10 min.

### Cell culture

MC3T3-E1 murine embryonic osteoblasts cells (ATCC, Manassas, VA, USA) were cultured in Minimum Essential Medium α (αMEM) (Gibco, Grand Island, NY, USA) supplemented with 10% fetal bovine serum (FBS) (Gibco, Grand Island, NY, USA) at 37℃ under 5% CO_2_. After reaching 70–80% confluence, cells were harvested for further experiments. In certain in vitro experiments, MC3T3-E1 cells were exposed to 10 ng/mL TNFα to stimulate the inflammatory microenvironment.

### Conditioned medium (CM) preparation

UCMSCs were seeded in 10-cm dishes (50, 100, 150, 200 × 10^4^ cells/dish, respectively), and MC3T3-E1 cells were seeded in 10-cm dishes (100 × 10^4^ cells/dish) with αMEM containing 10% FBS. After incubation for 48 h, the cell supernatant was collected as CM of UCMSCs (UCMSC-CM), while the medium culturing MC3T3-E1 cells was used as control CM. After being centrifuged to remove cell debris, the UCMSC-CM and control CM went through a sterile filtration by a 0.22 μm filter, harvested and stored at − 80 °C for further use.

### Cell viability assay

Cell Counting Kit-8 (CCK-8) test was applied to assess cell viability and proliferation (Beyotime, Shanghai, China). MC3T3-E1 cells (5 × 10^3^ cells/well) were seeded in 96-well plates and treated with UCMSC-CM collected from various seeding cell numbers. To determine whether UCMSC-CM could restore the proliferation of MC3T3-E1 cells, 10 ng/mL TNFα was added to various concentrations of UCMSC-CM for 24–48 h. Then the medium was substituted with 100 µL of fresh medium with 10 µL of CCK-8 reagent. After cultured for another 2 h in the dark, the absorbance was measured on a microplate reader (Bio-Rad, CA, United States) at 450 nm.

### Osteoblast differentiation analysis

MC3T3-E1 cells were seeded in 24-well plates (5 × 10^4^ cells/well). Upon reaching 70-80% confluence, the cells were switched to an osteoblast-induced medium (OIM) (CM supplemented with 10% FBS, 10 mM β-glycerophosphate and 50 µg/ml ascorbic acid) and replaced every 2 days. Meanwhile, 10 ng/mL TNFα were added to the medium in the TNFα and TNFα + UCMSC-CM groups, while control group were only cultured in control CM. After 14 days of culture, alkaline phosphatase (ALP) staining was measured by using a 1-Step™ NBT/BCIP Kit (Thermo Fisher Scientific, Waltham, Massachusetts, USA). After 21 days of culture, alizarin red staining (ARS) was performed using an Alizarin Red S Staining Kit (Beyotime, Shanghai, China).

### Cell apoptosis assays

Apoptotic cells were detected using a TUNEL assay kit (C1088, Beyotime, Shanghai, China) and a FITC Annexin V Apoptosis Detection Kit (556547, BD Pharmingen, NJ, USA). Briefly, after treatment, MC3T3-E1 cells seeded in 6-well plates were fixed in 4% PFA for 30 min following cultured for 10 min with 0.3% Trition X. Afterwards, the cells were cultured in TUNEL assay solution in dark for 1 h, followed by DAPI for 10 min. For flow cytometry analysis. The dead and live MC3T3-E1 cells were collected, centrifuged, washed, resuspended and incubated in 300 µL 1× binding buffer with 5 µL Annexin V-FITC and 5 µL PI and cultured for 15 min. After that, the apoptosis rate (%) was subsequently assessed by a flow cytometer (BD Accuri C6, NJ, United States).

### Quantitative real-time polymerase chain reaction (qRT-PCR)

The total RNA was extracted from MC3T3-E1 cells after 7 days of treatment using TRIzol reagent (Invitrogen, Carlsbad, CA, USA). cDNA was generated by PrimeScript RT Master Mix (TaKaRa, Otsu, Japan), and SYBR Premix EX Taq™ kit (Takara, Otsu, Japan) was used for PCR in a QuantStudioTM 7 Flex Real-Time PCR System (Thermo Fisher Scientific, MA, USA). The primer sequences for the genes were listed in Table 1. 2^−ΔΔCT^ was calculated for relative fold change to determine the gene expression of the target genes relative to β-actin.

**Table 1:** Mouse real-time PCR primer sequences used in this study

Gene	Forward primer (5 ′ -3 ′)	Reverse primer (5 ′ -3 ′)
*Ocn*	GCAATAAGGTAGTGAACAGACTCC	CCATAGATGCGTTTGTAGGCGG
*Osx*	GGCTTTTCTGCGGCAAGAGGTT	CGCTGATGTTTGCTCAAGTGGTC
*Runx2*	CCTGAACTCTGCACCAAGTCCT	TCATCTGGCTCAGATAGGAGGG
*β-actin*	CATTGCTGACAGGATGCAGAAGG	TGCTGGAAGGTGGACAGTGAGG

### Western blotting analysis

After 7 days of treatment, the cells were lysed in immunoprecipitation assay (RIPA) (CW Biotech, Beijing, China) buffer containing 1 mM phenylmethylsulfonyl fluoride and protease inhibitor cocktail (Cell Signaling Technology, Boston, USA) for 30 min. After centrifuging at 12,000 g for 15 min at 4 °C, the supernatant was collected. A Pierce™ BCA assay kit was used to determine protein concentration (Thermo Fisher Scientific, MA, USA). 10% SDS-PAGE was used to load and separate samples with equivalent concentrations (2 µg/µL) (Epizyme Biotech, Shanghai, China). The proteins were transferred onto nitrocellulose membranes (Millipore, MA, USA). After being blocked in 5% skim milk at room temperature for 1 h, the membranes were incubated with the primary antibodies against beta actin (β-actin; Proteintech, 20536-1-AP; 1:10000), alkaline phosphatase (Alp; Abcam, Arigo, ARG57422; 1:5000), runt-related transcription factor-2 (Runx2; Huabio, ET1612-47; 1:5000), Phospho-SMAD2 (pSmad2; Thermofisher, 44-244G; 1:1000) and Smad2 (CST, 5339 S; 1:1000) overnight at 4 °C. Thereafter, an HRP-conjugated secondary antibody was used for an hour. The protein bands were imaged using an LI-COR Odyssey^®^ scanner (LI‑COR Biosciences, Nebraska, USA). Quantitative analyses were calculated via ImageJ software (National Institutes of Health, Bethesda, MD, USA).

### Statistical analysis

Data management and analysis were performed using Prism 9 software (GraphPad software, San Diego, CA). Data are expressed as means ± SEM and analyzed by one-way or two-way analysis of variance (ANOVA) with Turkey’s test for multiple comparisons. A *P* value < 0.05 was considered statistically significant.

## Results

### Characterization of UCMSCs

The results of flow cytometry reported that the surface markers’ positive rates of CD29, CD90, CD34 and CD45 of UCMSCs were 99.99%, 98.09%, 1.75% and 0.02%, respectively, indicating a similar surface marker profile as MSCs (Fig. 1C) [22]. Following differentiation induction, trilineage commitment was confirmed, with cells developing osteogenic (mineralized nodules), adipogenic (lipid droplets), and chondrogenic (proteoglycans) phenotypes.

### SC injection of UCMSCs preserved cartilage structure and reduced OA severity in ACLT rats

**Fig. 2:**
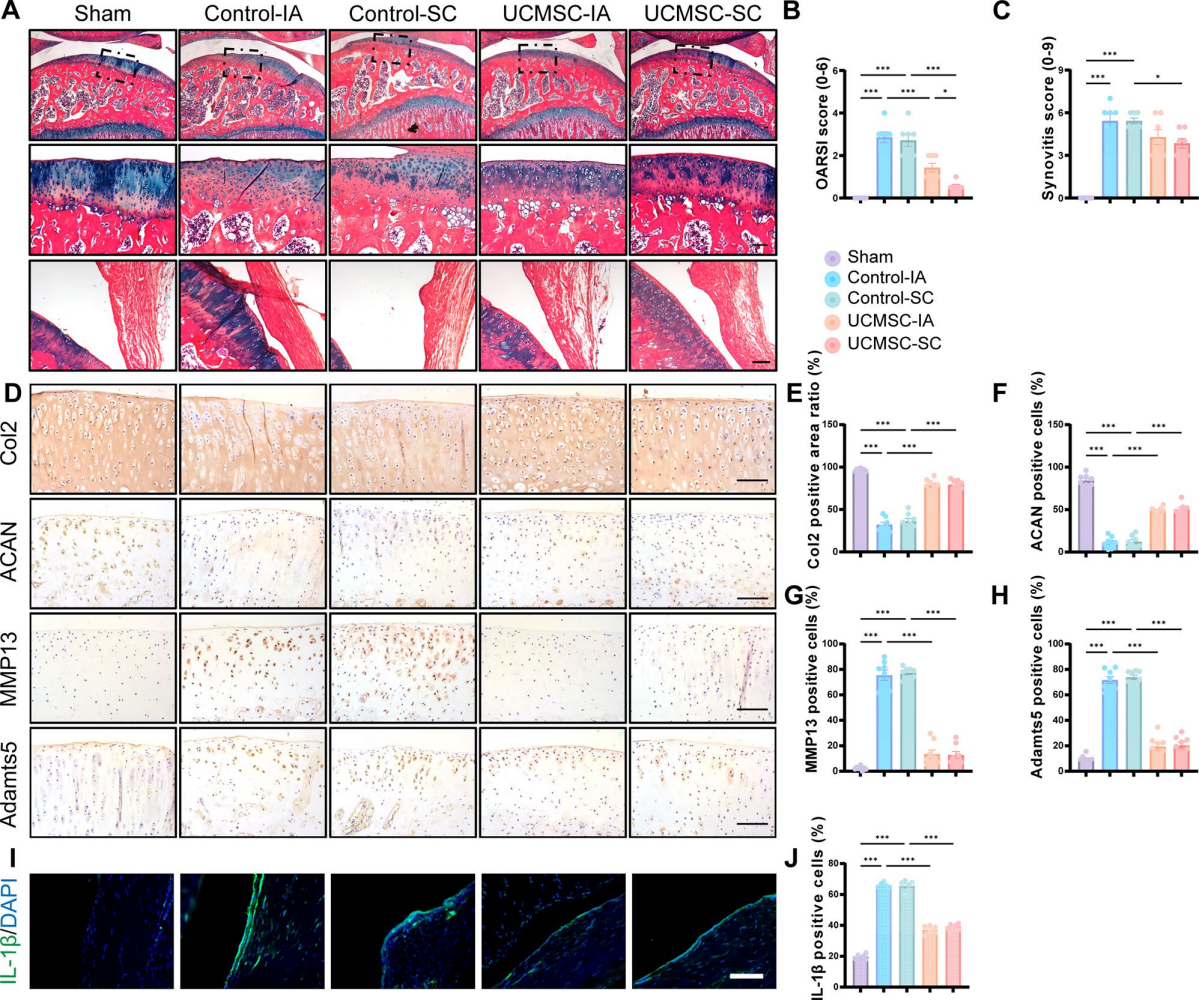
Intra-articular and subchondral bone injection of UCMSCs ameliorated OA progression in rat OA model. (**A**) Representative ABH/OG staining images of cartilage and synovium in different groups. Scale bar, 100 μm. (**B**-**C**) Quantitative analysis of the OARSI score and synovitis score. (**D**) Representative images of immunohistochemical (IHC) staining of different groups. Scale bar, 100 μm. (**E**-**H**) Quantitative analysis of the positive cells/area ratio of Col2, ACAN, MMP13 and adamts5. (**I**) Representative images of immunofluorescence (IF) staining of IL-1β in different groups. Scale bar, 100 μm. (**J**) Quantitative analysis of the positive cells of IL-1β. Data presented as means ± SEM by one-way ANOVA with Turkey’s post hoc test. **P < 0.01 and ***P < 0.001

ABH/OG staining was performed to investigate the histopathological features of articular cartilage (Fig. 2A). After ACLT, knee articular cartilage of control group exhibited severe cartilage degeneration including loss of proteoglycan and decrease of cartilage thickness, with increased OARSI scores of 2 at 8 weeks in control groups (Fig. 2B). In contrast, both intra-articular and subchondral bone injecting UCMSCs could preserved the cartilage with more abundant proteoglycan. OARSI scores were significantly different between intra-articular and subchondral bone treated ACLT rats. In line with previous study, intra-articular and inflammatory infiltration were seen in the control groups (Fig. 2A), which result in significantly higher synovitis scores than that of the sham controls (Fig. 2C). Besides, the IHC staining results indicated that UCMSCs administration by IA and SC significantly increased Col2 and ACAN (anabolism marker) expression while reduced MMP13 and adamts5 (catabolism marker) expression in articular cartilage of UCMSCs-treated ACLT rats relative to saline treated ACLT rats at 8 weeks (Fig. 2D-H). Furthermore, IF analysis demonstrated that both IA and SC administration of UCMSCs significantly reduced the expression of IL-1β (inflammatory marker) in the synovial tissue of ACLT rats compared to saline-treated controls (Fig. 2I, J). There was no significant difference with IA or SC treatments. These findings demonstrated that UCMSCs treatment by IA and SC could effectively restore articular cartilage homeostasis and inhibit synovial inflammation in post trauma OA models of rats.

### SC injection of UCMSCs alleviated ACLT-induced subchondral bone loss

**Fig. 3:**
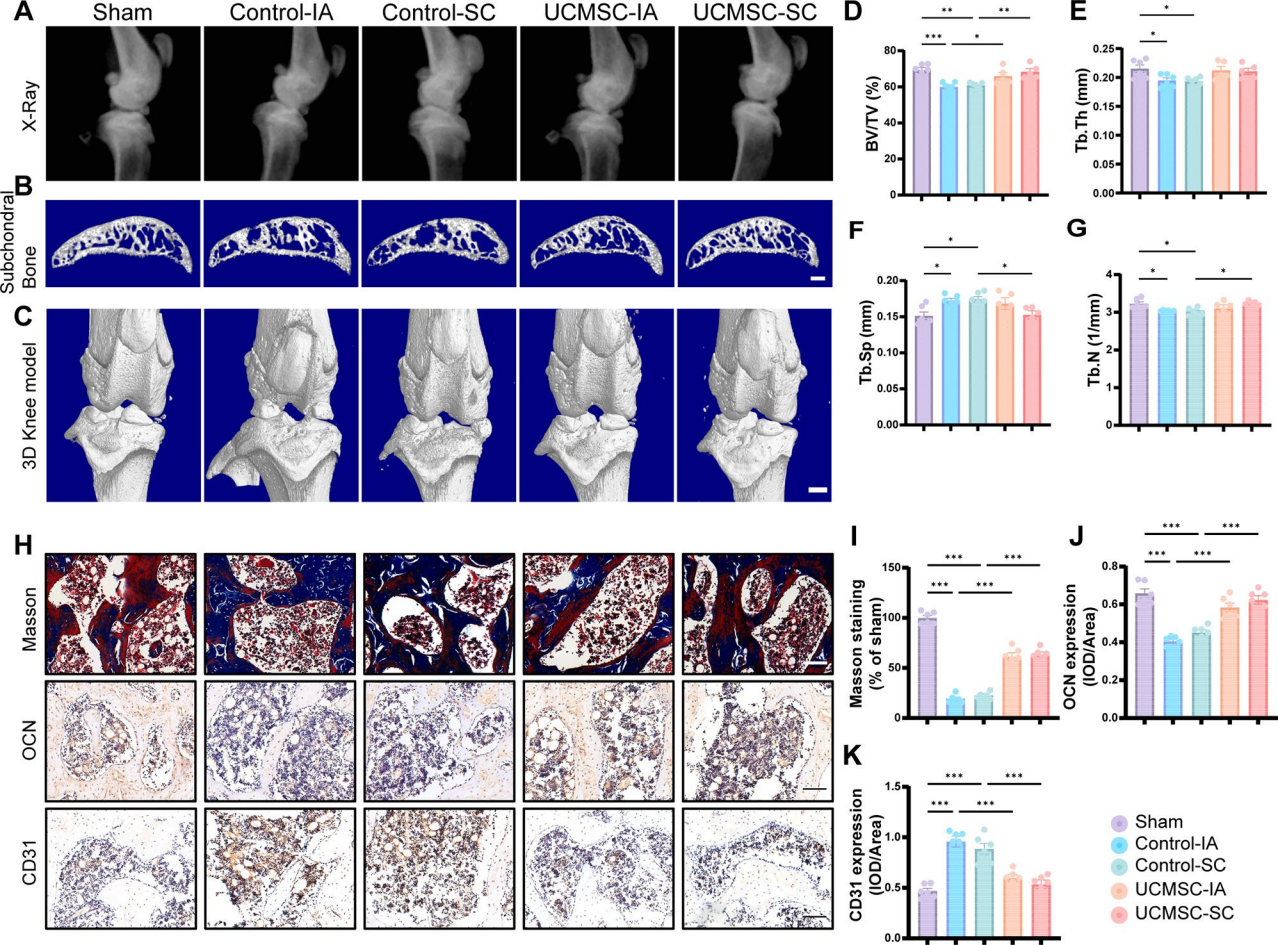
Intra-articular and subchondral bone injection of UCMSCs attenuated subchondral bone loss and enhanced bone formation in OA. (**A**) Representative sagittal X-Ray images of the knee joints of different groups. (**B**) Representative 3D reconstructed CT images of tibial subchondral bone of different groups. Scale bar, 250 μm. (**C**) 3D reconstructed CT images of knees of different groups. Scale bar, 1 mm. (**D**-**G**) Quantitative analysis of (**D**) BV/TV, (**E**) Tb.Th, (**F**) Tb.Sp and (**G**) Tb.N of each group. (**H**) Representative images of masson staining and IHC staining of different groups. (**I**) quantitative measurements of masson staining. (**J**-**K**) Quantitative analysis of the expression of OCN and CD31. Data presented as means ± SEM by one-way ANOVA with Turkey’s post hoc test. *P < 0.05, **P < 0.01 and ***P < 0.001

Next, we performed X-ray and µCT analysis to conduct radiological assessment of alterations in rat joints. X-ray results exhibited characteristic narrowing joint space and irregular contours of the articular surface in ACLT models, while IA or SC injection of UCMSCs significantly improved this pathological change (Fig. 3A). 3D reconstruction of tibial subchondral bone structures showed bone loss in the ACLT control groups, whereas UCMSCs treatment significantly ameliorated bone loss (Fig. 3B). The whole joint 3D reconstruction results showed marginal osteophytes and other alterations, indicating UCMSCs treatment could notably preserved the bone morphology (Fig. 3C). Furthermore, UCMSCs treatment by IA and SC both increased BV/TV and their Tb.Th was comparable to those observed in the sham group (Fig. 3D, E). Notably, only SC injection of UCMSCs significantly improved critical trabecular bone parameters, including increased Tb.N and decreased Tb.Sp. This suggests that although both administration routes attenuated bone loss compared to untreated controls, the SC approach exhibited more comprehensive preservation of trabecular architecture (Fig. 3F, G).

### SC injection of UCMSCs promoted osteoblastic bone formation and attenuated aberrant angiogenesis of subchondral bone in ACLT-induced OA rats

Bone formation in the tibial subchondral bone of rats was observed by Masson staining. The results indicated that UCMSCs-treated groups exhibited a higher percentage of new bone area relative to ACLT-induced rats (Fig. 3H, I). Moreover, the IHC of osteogenic and angiogenesis-associated proteins revealed results similar to the intra-articular-injected and subchondral bone-injected UCMSCs groups; both increased the expression of the osteogenic protein (OCN) while suppressing the expression of angiogenesis proteins (CD31) compared to control groups, indicating improved bone formation (Fig. 3H and J, K).

### SC injection of UCMSCs inhibited osteoblast apoptosis and Smad2-dependent TGF-β signaling pathway in ACLT-induced rat OA model

**Fig. 4:**
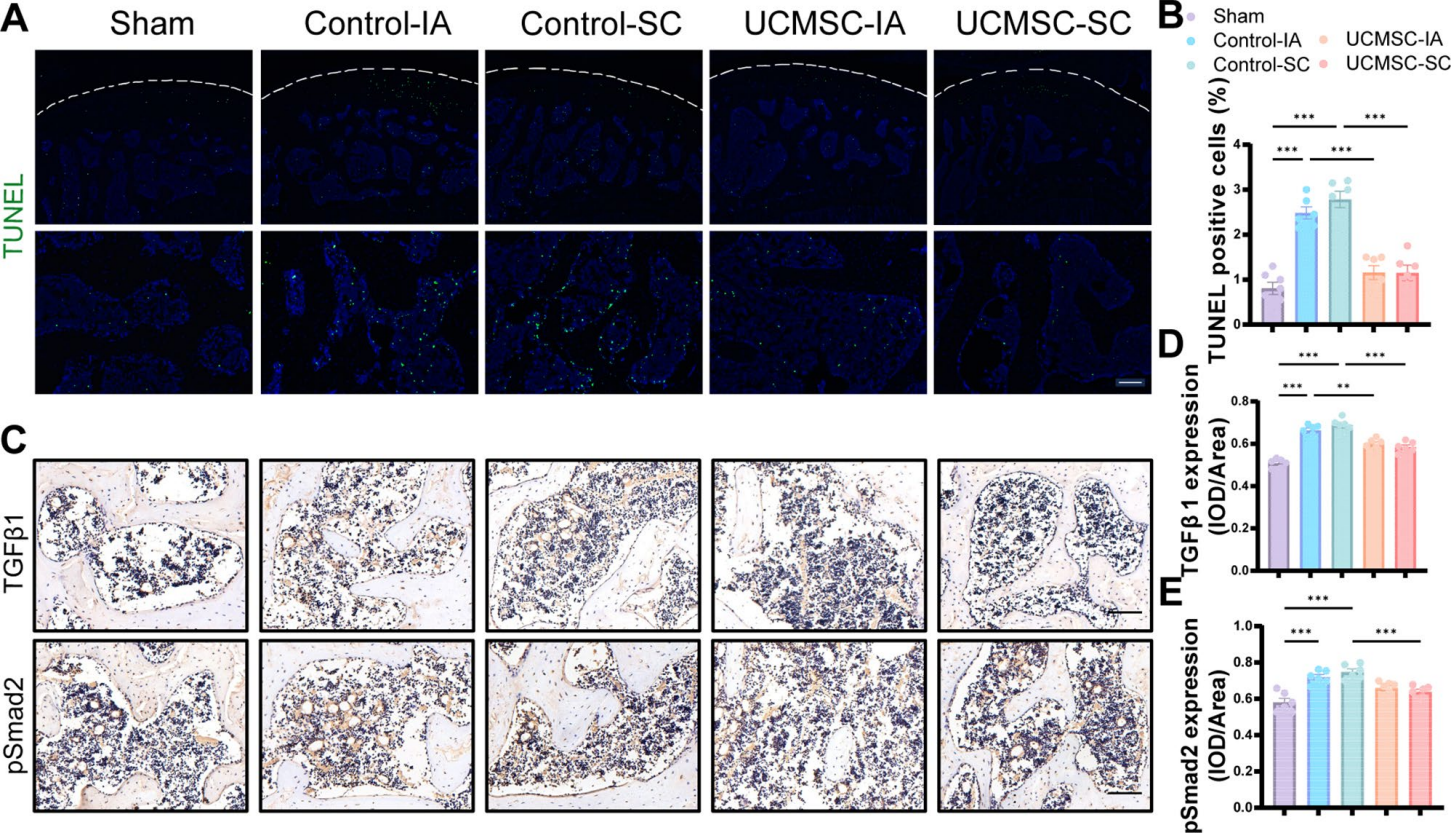
UCMSCs inhibited both the osteoblast apoptosis and the Smad2-dependent TGF-β signaling pathway in OA. (**A**) Representative images of TUNEL staining. Scale bar, 100 μm. (**B**) Quantitative analysis of the TUNEL positive cells. (**C**-**E**) IHC staining and quantitative analysis of TGFβ1 and pSmad2. Scale bar, 100 μm. Data presented as means ± SEM by one-way ANOVA with Turkey’s post hoc test. **P < 0.01 and ***P < 0.001

To explore the metabolic basis for impaired osteoblast activity in ACLT rats, we conducted TUNEL staining. The results exhibited significantly higher levels of apoptosis in the subchondral bone of control groups than in sham group. By contrast, this effect was attenuated by UCMSCs treatment via IA or SC (Fig. 4A, B). As high active TGF-β signaling in the subchondral bone initiate the pathological changes of OA, we investigated whether UCMSCs could directly inhibits TGF-β signaling in subchondral bone MSCs. Notably, The IHC staining validated the expression of TGFβ1 and pSmad2 were both elevated in the control groups relative to the sham group, whereas both the treatment of UCMSCs by IA and SC could rescue this growing trend and lower the expression of TGFβ1 (Fig. 4C, D). However, the significant decline of pSmad2 was only observed in the SC injected group (Fig. 4E).

### UCMSC-CM promoted osteoblast cell viability and counteracted osteogenic reduction effects of TNFα in vitro

**Fig. 5:**
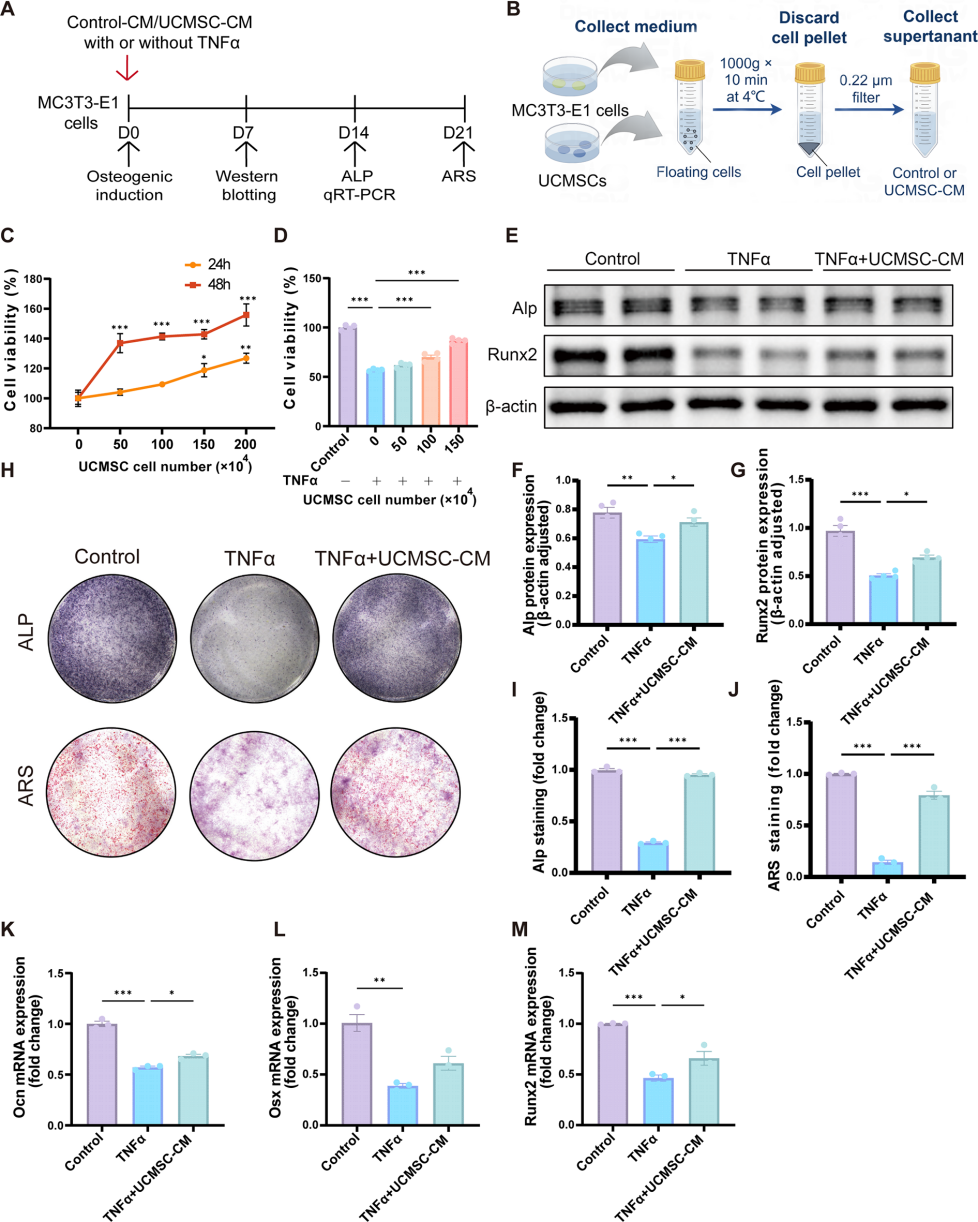
The effect of UCMSC-CM on the osteogenic differentiation of MC3T3-E1 cells. (**A**) UCMSC-CM treatment scheme. (**B**) The preparation process of UCMSC-CM. (**C**) The cell viability of MC3T3-E1 cells incubated with UCMSC-CM obtained from increasing seeding cell numbers for 24 and 48 h, versus 0 h group. (**D**) The viability of MC3T3-E1 cells treated with TNFα (10 ng/mL) plus UCMSC-CM by CCK-8 assay. (**E**-**G**) The protein expression of Alp and Runx2 in MC3T3-E1 cells treated with TNFα and UCMSC-CM. (**H**-**J**) Alkaline phosphatase (ALP) (top) and alizarin red (bottom) staining of MC3T3-E1 cells treated with TNFα and UCMSC-CM for 14 and 21 days, respectively. (**K**-**M**) The relative mRNA expression of *Ocn*, *Osx* and *Runx2* of MC3T3-E1 cells treated with TNFα and UCMSC-CM. Data presented as means ± SEM by one-way ANOVA with Turkey’s or Dunnett’s post hoc test. *P < 0.05, **P < 0.01, and ***P < 0.001

To examine the paracrine effects of UCMSCs on TNFα-induced osteoblasts, we adopted a CM system (Fig. 5A, B). The CCK8 assay was performed to assess the effects of UCMSC-CM on the cell viability of MC3T3-E1 cells. The results showed that UCMSC-CM derived from different cell densities significantly increased the cell viability of MC3T3-E1 cells after 24- and 48-h treatment (Fig. 5C). Notably, UCMSC-CM significantly enhanced the proliferation of MC3T3-E1 cells inhibited by TNFα (Fig. 5D). To explore the osteogenic effects of UCMSC-CM, MC3T3-E1 cells were induced by OIM with 10 ng/ml TNFα, with or without UCMSC-CM. The Western blotting results showed that UCMSC-CM treatment reversed the expression of Alp and Runx2 induced by TNFα (Fig. 5E-G). Furthermore, MC3T3-E1 cells exhibited decreased ALP activity and weak ARS after TNFα stimulation. This was restored after UCMSC-CM treatment, indicating that UCMSC-CM promoted cell osteogenic differentiation and mineralization (Fig. 5H-J). Consistent with our Western blotting results, qRT-PCR showed that the mRNA levels of *Ocn*, *Osx* and *Runx2* were markedly diminished after TNF-α stimulation compared with those in the control group, and it was ameliorated by UCMSC-CM treatment (Fig. 5K-M).

### UCMSC-CM inhibited osteoblast apoptosis and TGF-β signaling pathway in vitro

**Fig. 6:**
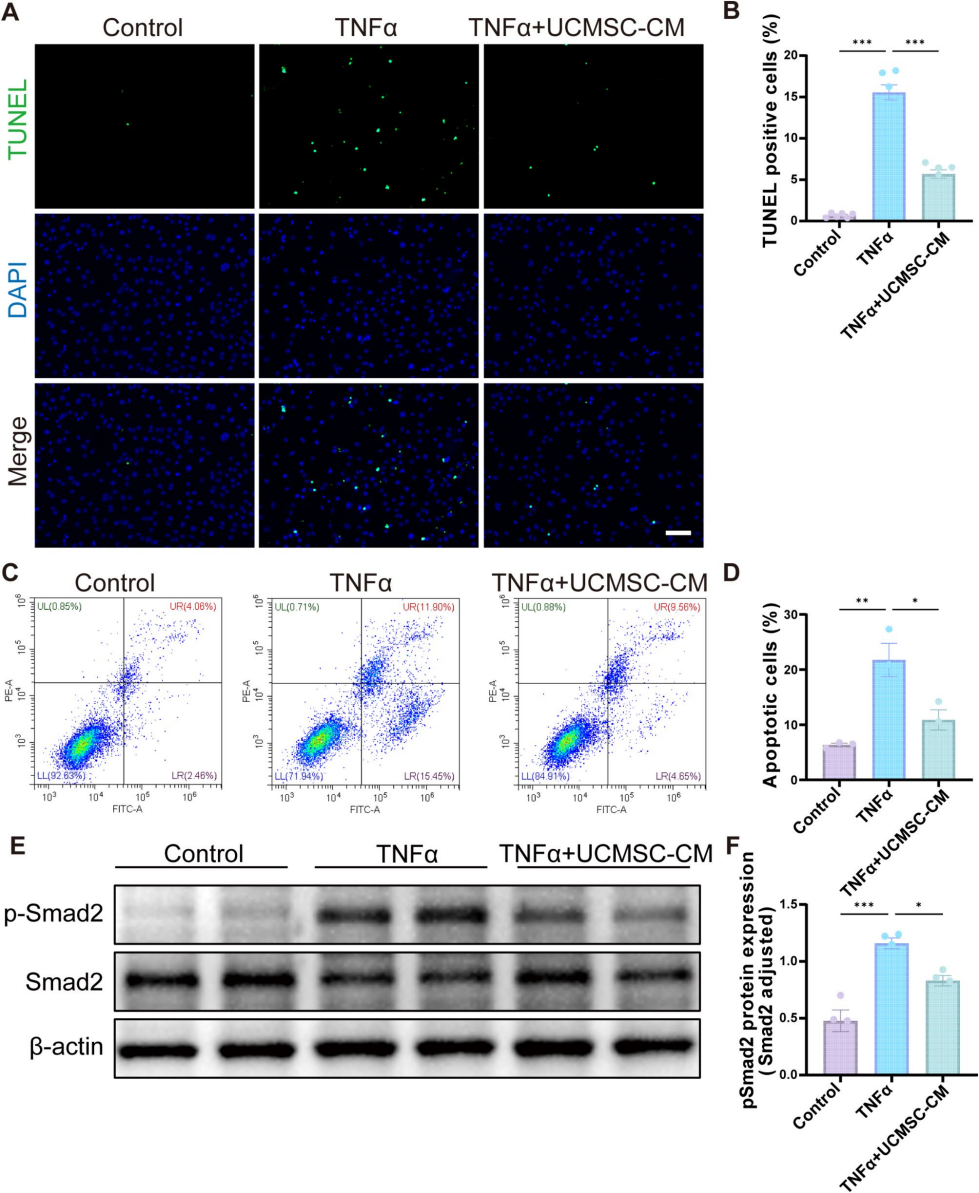
UCMSC-CM inhibited osteoblast apoptosis and TGF-β pathway of MC3T3-E1 cells. (A-B) TUNEL staining and quantitative analysis of MC3T3-E1 cells treated with TNFα and UCMSC-CM. Scale bar, 100 μm. (C-D) Flow cytometry analysis of MC3T3-E1cells after treatment with TNFα and UCMSC-CM. UL: necrosis cells, UR: late apoptotic cells, LL: viable cells and LR: early apoptotic cells. (E-F) The protein expression of p-Smad2 in MC3T3-E1 cells treated with TNFα and UCMSC-CM. Data presented as means ± SEM by one-way ANOVA with Turkey’s post hoc test. *P < 0.05, **P < 0.01, and ***P < 0.001

Subsequently, the effect of UCMSC-CM on TNFα-induced osteoblast apoptosis was detected. TUNEL staining and flow cytometry results consistently confirmed that UCMSC-CM treatment significantly suppress the level of apoptosis in MC3T3-E1 cells (Fig. 6A-D). We further detected whether UCMSC-CM affects TGF-β signaling in MC3T3-E1 cells. The Western blotting analysis indicated that UCMSC-CM significantly downregulated pSmad2 expression of MC3TE-E1 cells in the presence of TNFα (Fig. 6E, F). The results indicated UCMSC-CM inhibits apoptosis and TGF-β/pSmad2 signaling in osteoblast.

## Discussion

**Fig. 7:**
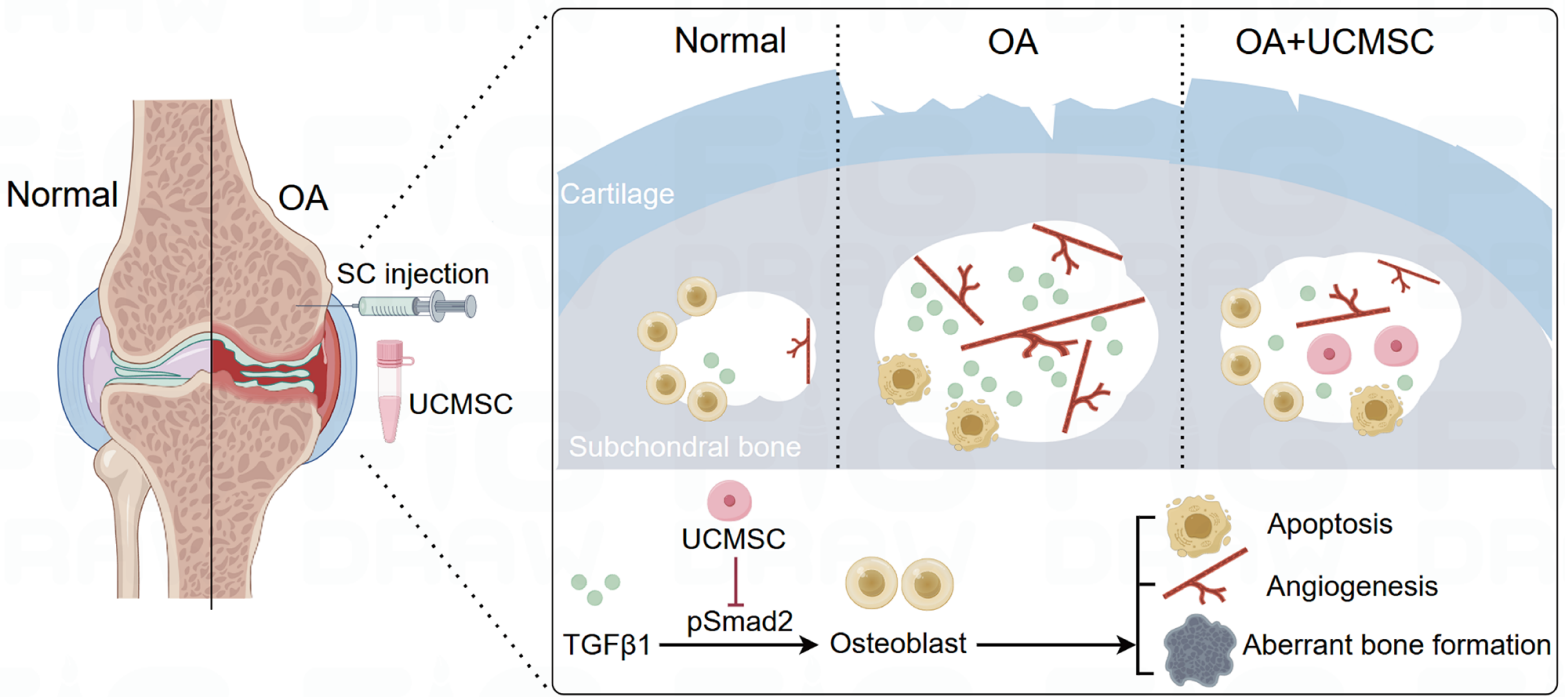
Therapeutic effects and underlying mechanisms of subchondral UCMSC injection on knee osteoarthritis

When it comes to OA, current therapeutic options are limited to drugs that provide only mild symptomatic benefit. One of the main obstacles is the complex pathological mechanism with the progression of OA and subchondral bone homeostasis is a major but often ignored problem in OA [39]. Growing evidence suggests that UCMSCs is an effective treatment for chronic degenerative diseases. Thus, in the present study, we investigate the efficacy of UCMSCs on OA in a rat ACLT model using different injection methods and explored the cellular and molecular mechanism of UCMSCs by applying TNFα-induced model of MC3T3-E1 cells. We observed that UCMSCs conserve the subchondral bone microarchitecture to manage OA by inhibition of ACLT-induced abnormal bone loss, angiogenesis as well as TNFα-induced osteoblast apoptosis, and excessive TGF-β activity in vivo and in vitro (Fig. 7). Comparative analysis revealed that SC injection of UCMSCs significantly improved subchondral bone parameters compared to OA controls, whereas IA delivery showed no such improvement. Both routes provided comparable cartilage protection; however, the superior OARSI histological outcomes in the SC group suggest a potential advantage in targeting integrated osteochondral pathology.

Articular cartilage and subchondral bone form a closely composited functional unit [40]. During OA, subchondral bone remodeling is occurred in response to mechanical loading, leading to dynamically alterations of the microarchitecture of subchondral bone, such as oscillated subchondral trabecular bone volume and subchondral bone plate thickness [1]. Afterwards, the hypomineralized subchondral bone with structure abnormalities may weaken structural support thus triggering the degradation of the overlying cartilage. Figure 3 demonstrates that both SC and IA injections of UCMSCs attenuated bone loss compared to the Control group. However, quantitative analysis revealed that only the SC approach achieved statistically significant preservation of trabecular bone microstructure parameters (*P* < 0.05). The superior structural protection observed in the SC group may stem from its direct modulation of the local microenvironment, whereas the IA route might exert more systemic effects. These results reflect those of Cui et al. [15] who also locally injected drug into subchondral bone in vivo and found that the pathogenesis of OA was effectively attenuated. Similarly, in another study, a single combined IA and SC injection of platelet-rich plasma (PRP) or bone marrow aspirate concentrate (BMAC) in monosodium iodoacetate (MIA)-induced OA rat knee led to improvement of cartilage and subchondral bone lesions, primarily due to decreased abnormal vascularization and remodeling while had no significant advantages in synovitis suppression [41]. Several studies of human OA also suggest subchondral bone as a target for pharmaceutical intervention [29, 30, 42]. For example, Hernigou et al. [42] demonstrated that SC injection of MSCs is more effective than IA injection to OA patients to ameliorate joint pain, improve MRI outcome and postpone TKA after fifteen years of follow-up. Besides, a combination of SC and IA injection of PRP or MSCs also led to satisfactory functional improvement and pain relief of OA patients [43]. These results might be related to rescued bone formation, cartilage turnover and subchondral MSCs proliferative and stress-resistance potentials after intraosseous infiltrations [41, 44]. Abnormal mechanical strain triggers dysregulated metabolism in osteoblasts, which is characterized by decreased expression of OCN. It is possible, therefore, that SC injection of UCMSCs may act on the osteogenic function of osteoblasts. In our research, both in vivo and in vitro evidence revealed that UCMSCs promote bone formation and osteogenic differentiation in osteoblast through paracrine-based actions, and likely prevents excessive angiogenesis. Consistent with the bone-cartilage crosstalk theory [12], the cartilage degeneration and metabolism was also ameliorated after treated with UCMSCs following the subchondral bone homeostasis reconstruction process.

Apoptosis is an evolutionarily conserved form of programmed cell death crucial for organismal development and homeostasis [45]. Apoptosis in osteoblast has been established as a major contributor to reduced bone mass and osteoporosis [46]. On the one hand, the balance of apoptosis and survival is essential to physiological bone turnover, repair, and regeneration in skeleton. However, the overactive apoptosis disrupts the balance of osteoblast proliferation and apoptosis, leading to declined osteoblast population, impaired differentiation and even bone loss [47]. Inhibiting osteoblast apoptosis could effectively protect bone formation. In line with this, we found osteoblast apoptosis were greatly upregulated induced by ACLT in vivo and TNFα in vitro, while this phenomenon was significantly reversed by UCMSCs treatment. This finding agrees with previous studies that UCMSCs were reported to be able to inhibit apoptosis in iodoacetic acid -induced OA [48]. On the other hand, the crosstalk between osteoblast and osteoclast lineage cells is considered as a key factor contributing to subchondral bone remodeling: biomechanical dysfunction will stimulate the release of RANKL and inhibit the production of OPG in osteoblasts due to elevated proinflammatory cytokines such as TNFα and IL-6, thus upregulating the expression of RANK in osteoclasts, a major contributor to excessive osteoclastogenesis and increased bone resorption activity [49]. Therefore, inhibiting apoptosis may effectively affect the OPG/RANKL/RANK system, another possible cause of subchondral bone loss. Further mechanistic studies regarding the role of UCMSCs on osteoblast and osteoclast crosstalk, and co-culture system such as tranwell assay are needed.

TGF-β signaling plays a pivotal role in maintaining homeostasis of both articular cartilage and subchondral bone in OA progression [50]. TGFβ1 expression was increased in human OA subchondral bone [51]. In addition, it was confirmed that inhibition of TGFβ1 could recover interrupted bone remodeling and angiogenesis as well as attenuated the degeneration of articular cartilage by inactivating Smad2/3 in the subchondral bone in ACLT-induced OA [52]. Meanwhile, Previous research has established that elevated apoptosis can specifically boosted TGF-β/Smad signaling pathway, and vice versa [53, 54]. Hence, it could conceivably be hypothesized that inhibition of this process could be a potential target for OA treatment. Notably, our results showed that SC and IA injection of UCMSCs could suppress pathological angiogenesis and hinder the TGF-β signaling in subchondral bone, which was in line with previous study [55]. Simultaneously, our in vitro data indicated that UCMSCs could inhibit Smad2 phosphorylation caused by TNF-α in MC3T3-E1 cells, further supporting that UCMSCs take therapeutic effect in a TGF-β signaling dependent manner, at least in osteoblasts.

Our study utilized the full ACLT model to recapitulate late-stage OA pathology, characterized by rapid cartilage degradation and subchondral bone remodeling, which aligns with its established role in evaluating interventions targeting advanced OA progression [56]. Notably, partial ACLT models induce gradual joint instability and milder OA phenotypes, serving as tools for early-stage OA research, whereas full ACLT triggers severe structural derangements akin to end-stage human OA [57, 58]. This distinction underscores that our findings primarily reflect UCMSC efficacy in a high-severity context. However, the inflammatory microenvironment in advanced OA may compromise UCMSC survival or paracrine activity, potentially limiting therapeutic outcomes compared to early interventions [59]. Future studies should employ progressive injury models (e.g., partial ACLT with timed UCMSC administration) to delineate stage-dependent efficacy and optimize treatment windows.

As far as we know, this study appears to be the first to compare SC with IA injection of UCMSCs in ACLT-induced OA rats and specifically investigate the underlying regulatory mechanisms. Our findings provide support for clinical application of UCMSCs using different injection methods. We found that both SC and IA injection of UCMSCs can attenuate OA progression, whereas SC injecting UCMSCs offered better protection against subchondral bone deterioration relative to IA injection. This discrepancy could be attributed to the most direct and immediate benefit of SC injection of UCMSCs to the subchondral bone site. Although SC played a more comprehensive role in treating OA, personalized solution should be adopted in different circumstances when cost-effectiveness and additional intoxicants are considered. Also noteworthy is that SC injection relies on radiographic guidance and the bone is difficult to penetrate. Thus, in clinical, SC injection might not be convenient for doctors and patients compared with IA injection. Unavoidably, this study has some limitations. First, the specific components of UCMSCs that exert their effects are still unknown, yet our in vitro results provide some tentative initial evidence that UCMSCs may function through a paracrine mechanism. Second, clinical studies indicated that patients treated with UCMSCs yield significant improvements in pain [60]. Unfortunately, we did not carry out pain assessment such as gait behavior and von Frey to determine the effect of UCMSCs on OA-related pains. Third, the condition of SC and IA injection cannot be directly simulated in vitro. However, the focus of in vitro research is on revealing mechanisms, and we provided in vivo evidence of the efficacy of these injection methods. Additionally, we cannot rule out the possibility that other worthwhile signaling pathways other than TGF-β/pSmad2 signaling involved in the modulation of more target cell populations of subchondral bone such as osteoclast. Hence, this is an important issue for future research. Furthermore, a dose-dependent evaluation of UCMSCs in a rat model was lack despite in vitro concentration gradient being set up. Further studies, which take these variables into account, will need to be undertaken.

## Conclusion

Taken together, this study for the first time demonstrated the efficacy of UCMSCs for treating OA rats via SC injection. Particularly, it can be substantiated that the anti-OA efficacy of SC injection was equivalent to that of IA injection while SC injection of UCMSCs played a more effective role in maintaining microarchitecture in subchondral bone. This intriguing finding may be related to the inhibition of aberrant vascularization, osteoblast apoptosis and TGF-β signaling, thereby enhancing osteogenic differentiation, and ultimately maintaining osteochondral homeostasis. These findings ascertain underlying mechanisms of UCMSCs responsible for treating OA and raise attractive prospect regarding the implementation of UCMSCs SC injections.

## Electronic supplementary material

Below is the link to the electronic supplementary material.

Supplementary Material 1

## Data Availability

All the data associated with our findings are available upon request to the corresponding author. Uncropped Western blottings for Figs. 5E and 6E are provided in Supplementary Fig. S1.

## Abbreviations

OA: Osteoarthritis
SC: Subchondral
MSCs: Mesenchymal Stem Cells
IA: Intra-Articular
UCMSCs: Human Umbilical Cord Mesenchymal Stem Cells
ACLT: Anterior Cruciate Ligament Transection
TdT: Terminal Deoxynucleotidyl Transferase
TUNEL: Terminal Deoxynucleotidyl Transferase (TdT)-Mediated dUTP Nick-End Labelling
CCK-8: Cell Counting Kit-8
ALP: Alkaline Phosphatase
ARS: Alizarin Red Staining
qRT-PCR: Quantitative Real-Time Polymerase Chain Reaction
BV/TV: Bone Volume Fraction
TGF-β: TGF-Beta
αMEM: Alpha Modified Eagle’s Medium
FBS: Fetal Bovine Serum
FACS: Fluorescence Activated Cell Sorting
SPF: Specific Pathogen-Free
µCT: Micro-CT
PFA: Paraformaldehyde
ROI: Region of Interest
Tb·N: Trabecular Number
Tb·Th: Trabecular Thickness
Tb·Sp: Trabecular Separation
EDTA: Ethylenediamine Tetra Acetic Acid
ABH/OG: Alcian Blue Hematoxylin/Orange G
IHC: Immunohistochemistry
Col2: Collagen II
ACAN: Aggrecan
OCN: Osteocalcin
TGFβ1: TGF beta 1
DAB: Diaminobenzidine
CM: Conditioned Medium
UCMSC-CM: CM of UCMSCs
OIM: Osteoblast-Induced Medium
β-actin: Beta Actin
Runx2: Runt-Related Transcription Factor-2
pSmad2: Phospho-SMAD2
PRP: Platelet-Rich Plasma
BMAC: Bone Marrow Aspirate Concentrate
MIA: Monosodium Iodoacetate
