# Supplementary figures and images for: Subchondral injection of human umbilical cord mesenchymal stem cells ameliorates knee osteoarthritis by inhibiting osteoblast apoptosis and TGF-beta activity
Source: Stem Cell Res Ther. 2025 May 9;16:235. doi: 10.1186/s13287-025-04366-7 (PMC12065343; doi:10.1186/s13287-025-04366-7)

**Fig. S1.** Uncropped Western blots for Fig. 5E, 6E.

**Fig. 5E**

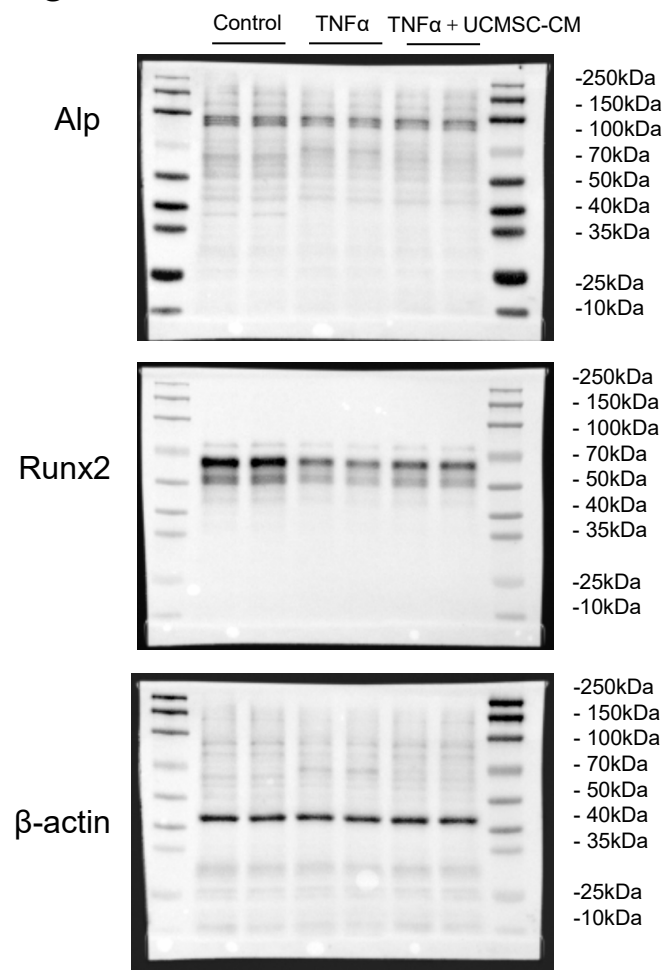

**Fig. 6E**

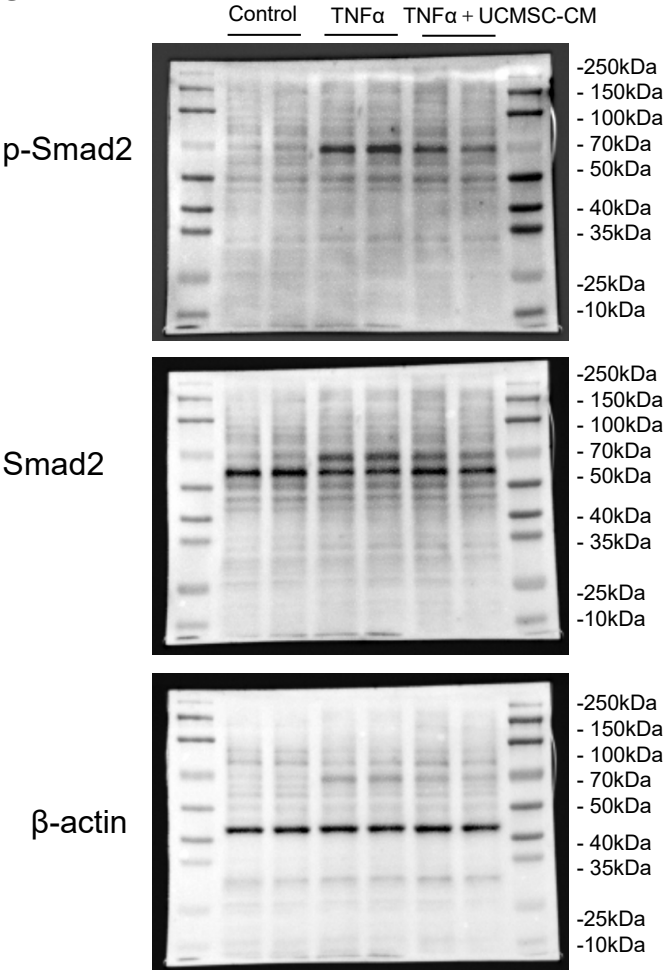

Supplement: Supplementary file 1 — Supplementary Material 1 [file 13287_2025_4366_MOESM1_ESM.pdf]
